# Comment on Campuzano-Donoso et al. Alcohol and Substance Use After Bariatric Surgery: Nutritional Risks and Clinical Implications in Long-Term Postoperative Care. *Nutrients* **2026**, *18*, 932

**DOI:** 10.3390/nu18213533

**Published:** 2026-10-27

**Authors:** Magnus Strømmen, Ragnhild Bergene Skråstad

**Affiliations:** 1 Centre for Obesity Research, Clinic of Surgery, St. Olavs Hospital, Trondheim University Hospital, 7006 Trondheim, Norway; 2 Faculty of Health Sciences and Social Care, Molde University College, 6410 Molde, Norway; 3 Department of Clinical and Molecular Medicine, Norwegian University of Science and Technology (NTNU), 7491 Trondheim, Norway; 4 Department of Clinical Pharmacology, St. Olavs Hospital, Trondheim University Hospital, 7006 Trondheim, Norway

The review by Campuzano-Donoso et al. is an important contribution, as it frames alcohol problems following bariatric surgery not only as an addiction issue, but also as a nutritional one [1]. In doing so, the authors highlight a clinically important *double vulnerability* which can be easily overlooked in practice: bariatric surgery itself predisposes to micronutrient deficiency, and alcohol use may further aggravate this risk. This perspective is highly relevant for long-term postoperative care. We also support the authors’ recommendation for more active screening of these patients. However, we are less convinced by the recommendation to use AUDIT-C for this purpose, particularly when it is presented as a validated screening tool in this clinical context.

AUDIT is the Alcohol Use Disorders Identification Test, originally developed as a brief screen for hazardous and harmful alcohol use in primary care settings. AUDIT-C refers to the first three items of AUDIT and only captures alcohol consumption. While AUDIT and AUDIT-C have been validated in several clinical populations and translated into multiple languages [2], the critical question here is the performance of AUDIT-C in the post-bariatric population.

This distinction matters because Roux-en-Y gastric bypass and sleeve gastrectomy substantially alter ethanol pharmacokinetics. Both procedures are associated with faster absorption, shorter time to peak concentration, and markedly higher peak blood alcohol concentrations [3,4,5]. Thus, the same reported alcohol intake may produce substantially greater systemic exposure after surgery. Campuzano-Donoso et al. summarize the literature well.

Accordingly, unchanged or only modestly changed AUDIT-C scores do not necessarily imply unchanged alcohol exposure or risk after bariatric surgery. To our knowledge, AUDIT-C has not been specifically validated or calibrated for the post-bariatric population. We therefore believe that uncritical use of AUDIT-C in this setting may yield false-negative or risk-underestimating results. Our concern is not that AUDIT-C lacks value in general, but that a purely consumption-based instrument may be insufficiently calibrated for a population with altered alcohol bioavailability and kinetics.

A more cautious clinical approach is therefore warranted. At present, self-reported alcohol consumption after bariatric surgery should be interpreted in light of the well-documented pharmacokinetic changes induced by these procedures. Ideally, modified interpretive thresholds, or a bariatric-specific adaptation of AUDIT-C, should be developed and validated. As a supplement, objective alcohol biomarkers such as phosphatidylethanol 16:0/18:1 (PEth) may have particular practical value. In recent years, several studies have explored the use of PEth in bariatric populations [6,7,8,9,10]. By providing an objective measure of recent alcohol exposure, PEth may help clinicians to anchor and individualize alcohol history, thereby facilitating conversations that can otherwise be difficult to conduct on the basis of self-report alone. Although important knowledge gaps remain, an objective marker of systemic alcohol exposure could be a valuable adjunct to self-reporting in this high-risk population.

## Data Availability

No new data were created or analyzed in this study. Data sharing is not applicable to this article.
